# *Notes from the Field:* Fatal Anthrax Pneumonia in Welders and Other Metalworkers Caused by *Bacillus cereus* Group Bacteria Containing Anthrax Toxin Genes — U.S. Gulf Coast States, 1994–2020

**DOI:** 10.15585/mmwr.mm7041a4

**Published:** 2021-10-15

**Authors:** Patrick Dawson, Caroline A. Schrodt, Karl Feldmann, Rita M. Traxler, Jay E. Gee, Cari B. Kolton, Chung K. Marston, Christopher A. Gulvik, James M. Antonini, María E. Negrón, John R. McQuiston, Kate Hendricks, Zachary Weiner, Gary A. Balsamo, Theresa Sokol, Paul Byers, Kathryn Taylor, Saad Zaheer, Stephen Long, Briana O’Sullivan, Marie A. de Perio, Alex R. Hoffmaster, Johanna S. Salzer, William A. Bower

**Affiliations:** ^1^Division of High-Consequence Pathogens and Pathology, National Center for Emerging and Zoonotic Infectious Diseases, CDC; ^2^Epidemic Intelligence Service, CDC; ^3^National Institute for Occupational Safety and Health, CDC; ^4^Louisiana Department of Health; ^5^Mississippi State Department of Health; ^6^Harris County Public Health, Houston, Texas; ^7^Houston Health Department, Houston, Texas; ^8^Texas Department of State Health Services, Austin, Texas.

In 2020, CDC confirmed two cases of pneumonia (one fatal) in welders caused by rare *Bacillus cereus* group bacteria containing anthrax toxin genes typically associated with *Bacillus anthracis*. *B. cereus* group bacteria are gram-positive facultative anaerobes, often toxin-producing, that are ubiquitous in the environment and reside naturally in soil and dust (*1*). *B. cereus* can also be found in food, and although infection typically causes illnesses characterized by diarrhea or vomiting, *B. cereus* can have other clinical manifestations (e.g., pulmonary, ocular, or cutaneous). Among seven persons in the United States reported to be infected with *B. cereus* group bacteria containing anthrax toxin genes resulting in pneumonia since 1994, five patients died and two had critical illness with prolonged hospitalization and recovery (*2*–*5*). All persons with pneumonia were welders or other metalworkers who had worked in Louisiana or Texas (Table). In addition to the seven pneumonia cases, a cutaneous infection with *B. cereus* group bacteria containing anthrax toxin genes has been reported in a patient with an anthrax eschar in Florida.^†^

**TABLE:** Characteristics of patients with severe anthrax pneumonia caused by non–*Bacillus anthracis* infections* with *Bacillus cereus* group bacteria containing anthrax toxin genes — Louisiana and Texas, 1994–2020

Patient	Year	Work location	Strain^†^	Age, yrs	Sex	Occupation	Outcome
A^§^	1994^¶^	Louisiana	*B. cereus* G9241**	42	Male	Welder^††^	Recovered
B^§^	2003	Texas	*B. cereus* 03BB87**	56	Male	Metalworker	Died
C^§^	2003	Texas	*B. cereus* 03BB102	39	Male	Welder	Died
D^§§^	2007	Louisiana	*B. cereus* LA2007**	47	Female	Metalworker	Died
E^¶¶^	2011	Texas	*B. cereus* Elc2	39	Male	Welder	Died
F	2020	Louisiana***	*B. cereus* LA2020**	39	Male	Welder	Recovered^†††^
G	2020	Texas	*B. cereus* TX2020	34	Male	Welder	Died

* Excludes laboratory-acquired infections.

^†^ Strain names are from original reported designations. Previous strain name assignments might not reflect current classifications.

^§^ https://academic.oup.com/cid/article/44/3/414/314305

^¶^ The 1994 case was retrospectively identified following the reports of the 2003 cases, prompting a review of *Bacillus* isolates that caused unusually severe disease. https://www.pnas.org/content/101/22/8449

** Recent taxonomic updates have subdivided the *B. cereus* group into an additional nine species (https://www.microbiologyresearch.org/content/journal/ijsem/10.1099/ijsem.0.001821). Whole genome sequence analysis suggests these isolates would be classified as newly described *Bacillus tropicus*.

^††^ Welders, a subset of metalworkers, are listed separately because five of the seven patients were welders.

^§§^ https://journals.asm.org/doi/10.1128/genomeA.00181-17

^¶¶^ https://meridian.allenpress.com/aplm/article/135/11/1447/64984/

*** Patient F is a Mississippi resident who had recently worked as a welder in Louisiana. An investigation by CDC at patient F’s worksite in Louisiana identified a bacterial isolate in a soil sample that genetically matched a clinical isolate from patient F.

††† Patient F received anthrax antitoxin.

Understanding the extent to which *Bacillus* species other than *B. anthracis* carry anthrax toxin genes and whether their geographic range extends beyond the U.S. Gulf Coast states is limited. Furthermore, little is known about why these highly fatal pneumonia cases have only been detected among welders and other metalworkers. Long-term exposure to welding and metalworking fumes is associated with various forms of lung injury that can cause changes in lung function and increase susceptibility to pulmonary infections, including fatal pneumonia.^§^ An investigation by CDC at one patient’s worksite in Louisiana (patient F) identified a bacterial isolate in a soil sample that genetically matched a clinical isolate from the patient. However, it is unclear why only one person at each worksite became ill and what environmental or host risk factors might have facilitated the exposure and infection.

Several actions can decrease risk for lung injury or infection, including anthrax pneumonia caused by *B. cereus* group bacteria, among welders and other metalworkers. Because of the association between welding or metalworking and pulmonary infections or injury, it is important that employers educate workers regarding hazards associated with welding and measures they can take to minimize potential exposures. Welding and metalworking employers, trade associations, and unions might consider targeted outreach to increase workers’ awareness about pulmonary infections, including anthrax, especially those workers in the U.S. Gulf Coast states. In addition, employers should conduct a hazard assessment at worksites and consider the use of National Institute for Occupational Safety and Health–approved respirators as part of a written respiratory protection program.^¶^^,^**

Clinicians should consider *B. cereus* group bacteria in the differential diagnosis when treating welders and other metalworkers with severe, rapidly progressive pneumonia or other anthrax-like disease.^††^ *B. cereus* group bacteria identified on culture are not always contaminants; when *B. cereus* with anthrax toxin is suspected, laboratorians and clinicians should pursue additional testing through their state Laboratory Response Network laboratory.^§§^ Regional health departments and the Laboratory Response Network serve pivotal roles in pathogen detection and procuring anthrax antitoxin for confirmed cases.

## Footnotes

* These authors contributed equally to this report.

†
https://journals.plos.org/plosone/article?id=10.1371/journal.pone.0156987

§ https://www.sjweh.fi/article/625; https://www.tandfonline.com/doi/abs/10.1080/713611032

¶ Occupational Safety and Health Administration. 29 CFR Sect. 1910.134. https://www.osha.gov/enforcement/directives/cpl-02-02-054

**
https://www.cdc.gov/niosh/npptl/topics/respirators/disp_part/respsource.html

††
https://www.cdc.gov/anthrax/treatment/index.html

§§
https://emergency.cdc.gov/lrn/
